# Liver transient elastography values in healthy South African children

**DOI:** 10.1186/s12887-023-04170-3

**Published:** 2023-07-13

**Authors:** Penelope C. Rose, Mark F. Cotton, Kennedy Otwombe, Steve Innes, Etienne D. Nel

**Affiliations:** 1 Department of Paediatrics and Child Health, Tygerberg Hospital and Stellenbosch University, PO Box 241, Cape Town, 8000 South Africa; 2 Family Center for Research with Ubuntu (FAMCRU), Cape Town, South Africa; 3 Perinatal HIV Research Unit, School of Public Health, Chris Hani Baragwanath Academic Hospital, University of the Witwatersrand, Johannesburg, South Africa; 4 Desmond Tutu HIV Centre, University of Cape Town, Cape Town, South Africa

**Keywords:** Liver, Fibrosis, Paediatric, Hepatic, Normal

## Abstract

**Background:**

Transient elastography (TE) is a rapid noninvasive ultrasound-based technology that measures liver stiffness as a surrogate for liver fibrosis and controlled attenuation parameter (CAP) as a measure of liver steatosis. However, normal ranges in children are not well defined in all populations. The aim of this study was to determine transient elastography values in healthy South African children.

**Methods:**

From April 2019 to December 2021, children were recruited from the HIV negative control group of a cohort study. Only children neither overweight nor obese, without evidence of liver disease, no medical condition or medication associated with hepatic steatosis or fibrosis and normal metabolic profile were included in this cross-sectional analysis. Clinical data, anthropometry and blood samples were collected on the same day as transient elastography with controlled attenuation parameter was performed.

**Results:**

104 children (median age 12.8 years [IQR 11.4–14.8, range 7.9–17.7 years]; 59 [57%] boys) were included. Liver stiffness was positively correlated with age (Pearson’s r = 0.39, p < 0.001). Median liver stiffness in boys (5.2 kPa [5th to 95th percentiles 3.6 to 6.8 kPa]) was greater than in girls (4.6 kPa [5th to 95th percentiles 3.6 to 6.1 kPa; p = 0.004]), but there was no difference by ethnicity. Median CAP was 179dB/m (5th to 95th percentiles 158 to 233dB/m). There was a positive correlation between CAP and body mass index (BMI) z-score, but no difference by age, sex, ethnicity or pubertal status.

**Conclusion:**

Liver stiffness values increase with age and are higher in healthy South African boys than girls, whereas CAP values vary with BMI, but not with age or sex.

## Background

Although traditionally considered the gold standard in evaluating paediatric liver disease, liver biopsy is invasive, has complications and is unsuitable for routine monitoring of liver fibrosis or steatosis progression [1, 2]. A number of non-invasive methods have been developed to evaluate liver fibrosis. These include scores using either routinely available laboratory test results such as the aspartate transaminase-to-platelet ratio (APRI) or fibrosis-4 test or biological markers of fibrosis such as the Fibrotest or Enhanced Liver Fibrosis panel. Imaging-based techniques include transient elastography and acoustic radiation force impulse [3].

Transient elastography (TE) is a rapid non-invasive ultrasound-based technology using low-frequency elastic shear waves to measure liver stiffness as a surrogate for liver fibrosis. Controlled attenuation parameter (CAP) can be measured to quantify hepatic steatosis based on ultrasound signal attenuation [4]. TE has performed well in identifying children with significant liver fibrosis and is considered the method of choice to monitor the longitudinal progression of liver fibrosis in children [5–8]. Although commonly used in adults, there is still lack of clarity regarding the normal ranges and optimal cut-offs to identify steatosis and fibrosis in children [4, 5, 9–12]. In a recent meta-analysis, normal liver stiffness values in of largely healthy Caucasian and Asian children were described, with a lack of data from African and Hispanic populations [13].

The aims of this study were to describe liver stiffness and CAP values using transient elastography in healthy school-aged South African children without evidence of liver disease or metabolic risk factors for hepatic steatosis and also to determine factors influencing liver stiffness and CAP.

## Methods

### Study design, setting and participants

This was a cross-sectional substudy of an existing cohort of children at the Family Center for Research with Ubuntu (FAMCRU) at Tygerberg Hospital, Cape Town, South Africa, followed for early evidence of chronic non-communicable diseases. Children were recruited as HIV-uninfected controls from local communities after birth or during the first years of life and participated in either a vaccine trial linked to the Children with HIV Early antiRetroviral (CHER) trial or the P1104s neurocognitive substudy following an antiretroviral strategy trial [14, 15]. Children’s perinatal HIV exposure status was recorded and HIV negative status was confirmed using HIV rapid testing at enrolment into the parent cohort. Participants were consecutively enrolled at their routine study visits between April 2019 and December 2021.

For this substudy, children were excluded if they were overweight or obese with a body mass index (BMI) z-score over + 1, had known liver disease or any evidence of liver disease (alanine transaminase [ALT] above the upper limit of normal for age and sex or APRI > 0.5), any medical condition or medication associated with hepatic steatosis or fibrosis, consumed alcohol regularly or excessively (more than 2 units per day for females or 3 units per day for males) or had any metabolic risk factors, including elevated serum triglyceride (> 1.7mmol/L), low serum high-density lipoprotein (HDL) cholesterol (< 1.1mmol/L) or impaired fasting glucose (≥ 5.6mmol/L) [16]. All children were confirmed to be clinically well on the day of their study visit.

### Clinical data

All clinical data and blood samples were collected on the same day. Comorbid medical diagnoses and current or recent medications were documented from structured interview and medical record review. Anthropometric data included weight measured on an electronic scale in minimal clothing to the nearest 0.1 kg; height measured standing upright without shoes using a stadiometer to the nearest 0.1 cm; waist circumference measured in centimetres midway between the superior border of the iliac crest and the lowermost rib margin at end expiration to the nearest 0.1 cm; and thoracic perimeter measured at the xiphisternum at end expiration to the nearest 0.1 cm. Body mass index (BMI) was calculated as weight divided by height squared and the z-score was calculated using World Health Organization software (WHO AnthroPlus: https://www.who.int/growthref/tools/en/) [17]. Children were classified as being lean, overweight or obese if the BMI z-score was below + 1, +1 to + 2 or greater than + 2 respectively [18]. ALT, aspartate transaminase (AST), platelet count, triglycerides, cholesterol [total, HDL and low-density lipoprotein (LDL)] and glucose were assayed at the National Health Laboratory Service (NHLS), Tygerberg Hospital, after an overnight fast. The APRI was calculated as [(AST/upper limit of the normal AST range for age and gender) X 100]/platelet count.

### Transient elastography and controlled attenuation parameter

**Fig. 1:**
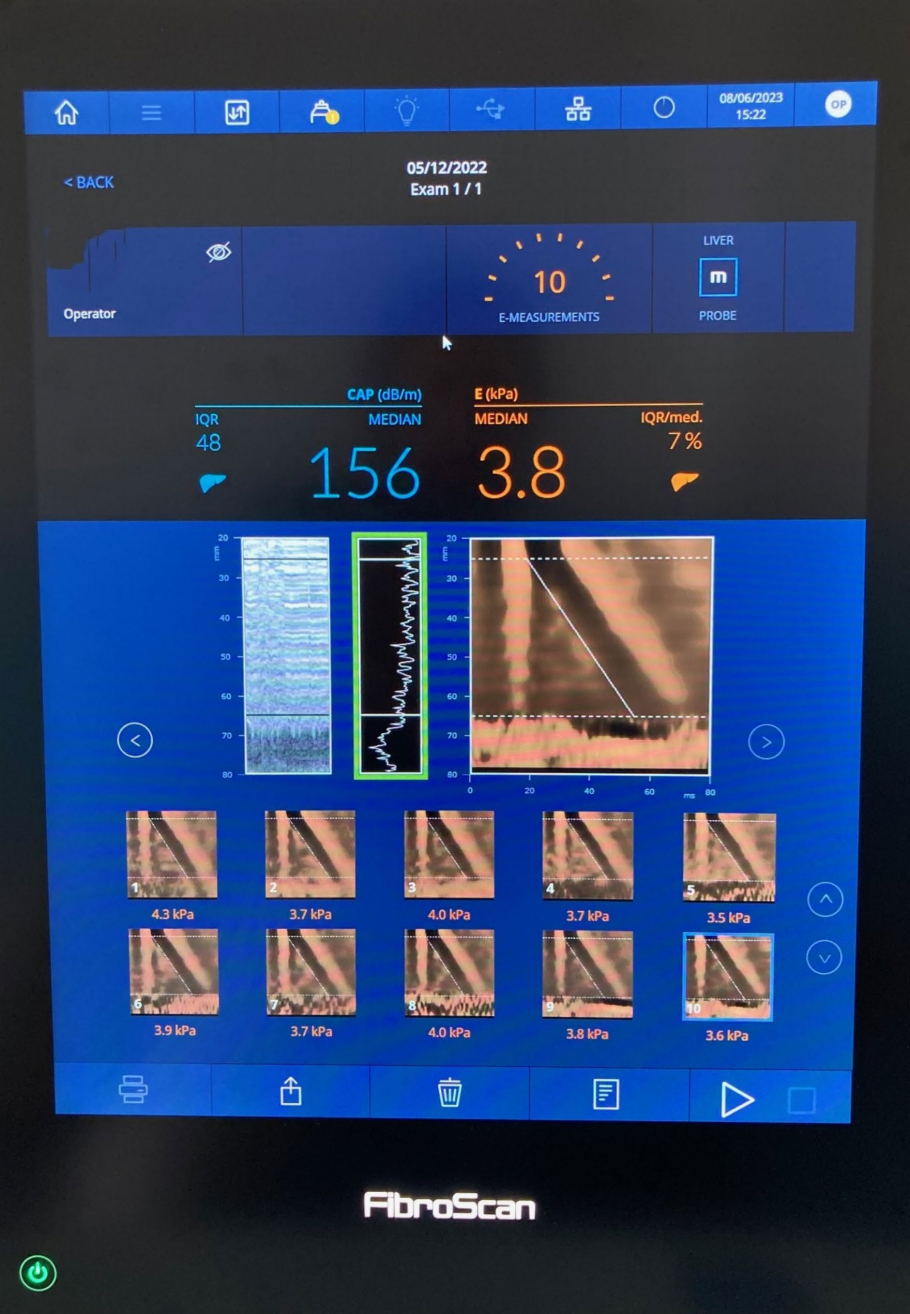
Transient elastography measurement

All children had liver stiffness and CAP measured after an overnight fast and were confirmed to be well on the day of evaluation, with no intercurrent illness. The FibroScan® uses vibration-controlled TE to assess liver stiffness as a measure of liver fibrosis. If the device has the necessary software installed, CAP can be measured simultaneously as a measure of hepatic steatosis. Two trained experienced operators measured TE by FibroScan® in all children using a standard M probe (diameter 7 mm). A scan was classified as successful if ten measurements were obtained with > 60% of attempted measurements producing a reading and if the liver stiffness interquartile range/median was < 30% [19]. The final result was the median measurement calculated by the Fibroscan® (Fig. 1). Results from unsuccessful scans were excluded from the analysis.

### Statistical methods

Descriptive results were expressed as frequencies and percentages for categorical variables and medians and interquartile ranges for continuous variables. Chi-square or Fisher’s exact test were used to compare categorical variables as appropriate, and t-tests or Wilcoxon rank sum tests for normally or non-normally distributed continuous variables respectively. The Shapiro-Wilk test was used to evaluate the normality of data distribution. Scatter diagrams were used to depict the distribution of liver stiffness by age. Pearson test evaluated correlations between normally-distributed continuous variables, and Spearman test where normality assumptions were not borne out. All statistical tests of significance were two-sided, with p < 0.05 considered statistically significant. Statistical analysis was performed using STATA version 12 (StataCorp LP, College Station, Texas, USA).

Ethical approval was obtained from the Stellenbosch University Health Research Ethics Committee (N12/11/076 and S20/02/046). Written informed consent was obtained from the parent or legal guardian and assent from all child participants.

## Results

### Clinical characteristics

**Fig. 2:**
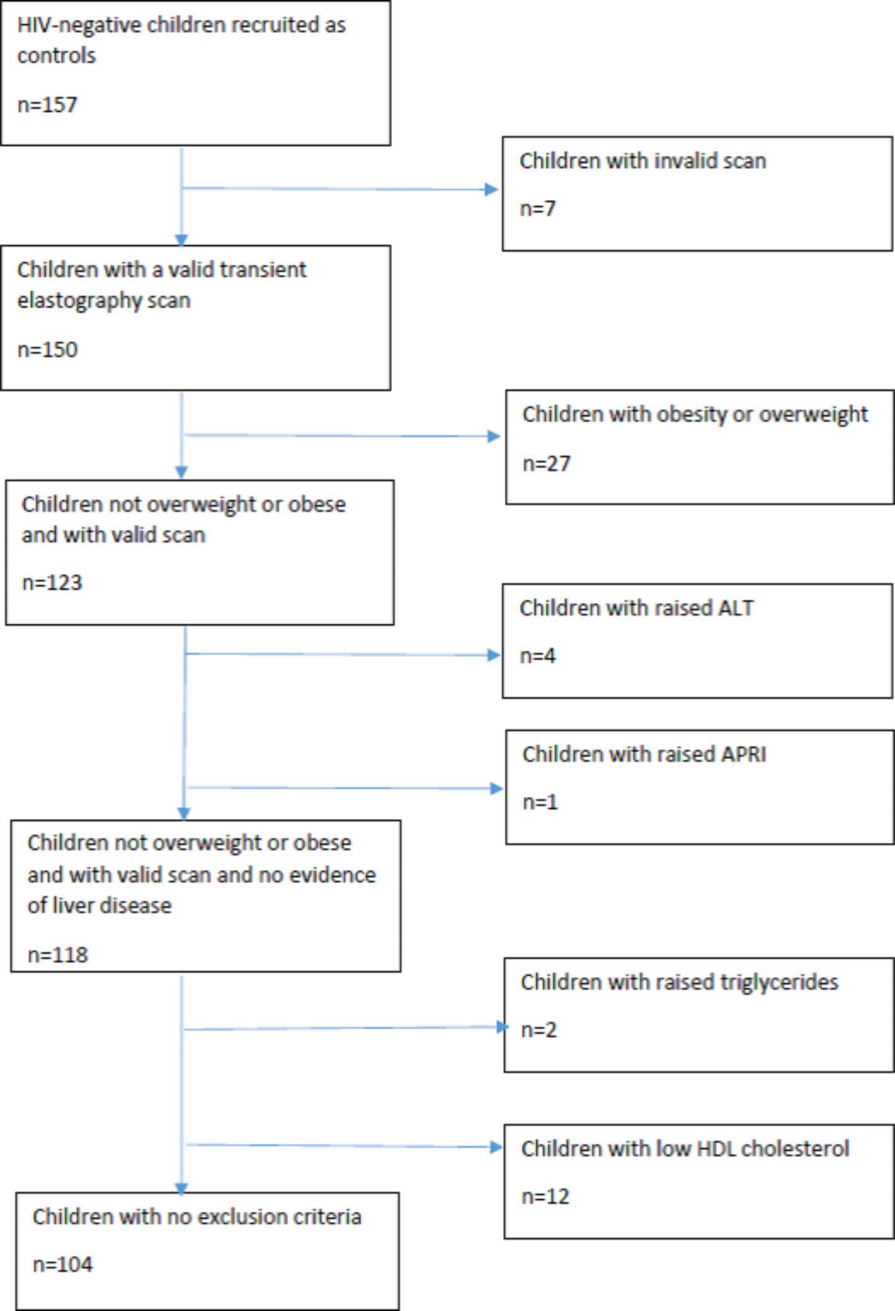
Flow chart of study recruitment

During the study period, 157 HIV-negative children ranging in age from 7 to 17 years were prospectively recruited and evaluated, with 150 (96%) having a reliable TE result using the M probe. All children were clinically well on the day of their study visit. TE using the M probe was well tolerated by all children. Eleven (7%) children reported ever consuming alcohol, but none met exclusion critieria. One hundred and four children (69%) were eligible and included in this sub-analysis (Fig. 2). The median age was 12.8 years [IQR 11.4–14.8 years], 59 (57%) were boys. Boys had higher waist-hip ratio and lower fasting HDL cholesterol than girls. Clinical characteristics, anthropometry and laboratory results are provided in Table 1.

**Table 1:** Clinical characteristics, anthropometry, laboratory results and transient elastography results of boys and girls

	All children n = 104	Boys n = 59	Girls n = 45	P-value
Age, years	12.8 (11.4–14.8)	13.2 (11.4–15.2)	12.6 (11.2–15.2)	0.3
Ethnicity				
African	70 (67%)	43 (73%)	27 (60%)	0.2
Mixed	34 (33%)	16 (27%)	18 (40%)	
Puberty				
Prepubertal	18 (17%)	7 (16%)	11 (19%)	0.7
Pubertal	86 (83%)	38 (84%)	48 (81%)	
HIV exposed un-infected	46 (44%)	26 (44%)	20 (44%)	1.0
HIV unexposed	58 (56%)	33 (56%)	25 (56%)	
Body mass index z-score	-0.33 (-0.96–0.34)	-0.4 (-1.26–0.11)	-0.17 (-0.72–0.39)	0.09
Waist circumference (cm)	63.6 (60.4–69.1)	64.0 (60.4–69.1)	63.6 (59.5–69.5)	0.5
Waist-hip ratio	0.80 (0.77–0.83)	0.81 (0.79–0.85)	0.78 (0.76–0.81)	0.0002
Fasting glucose (mmol/L)	4.5 (4.4–4.8)	4.6 (4.3–4.9)	4.5 (4.4–4.7)	0.8
Fasting triglycerides				
(mmol/L)	0.56 (0.45–0.72)	0.58 (0.43–0.71)	0.54 (0.46–0.72)	0.9
Fasting total cholesterol				
(mmol/L)	3.8 (3.3–4.3)	3.8 (3.3–4.2)	3.9 (3.4–4.4)	0.2
Fasting HDL cholesterol				
(mmol/L)	1.4 (1.3–1.7)	1.4 (1.2–1.6)	1.5 (1.4–1.7)	0.03
Fasting LDL cholesterol				
(mmol/L)	2.1 (1.7–2.5)	2.0 (1.7–2.5)	2.1 (1.7–2.6)	0.6
Fasting glucose (mmol/L)	4.5 (4.4–4.8)	4.6 (4.3–4.9)	4.5 (4.4–4.7)	1.0
ALT (u/L)	12 (10–15)	12 (11–15)	11 (9–15)	0.09
APRI	0.20 (0.16–0.25)	0.21 (0.16–0.25)	0.18 (0.15–0.24)	0.3

All continuous variables are presented as medians and interquartiles ranges

### Liver stiffness results

Median liver stiffness in boys was 5.2 kPa (5th to 95th percentiles 3.6 to 6.8 kPa), significantly greater than in girls (4.6 kPa (5th to 95th percentiles 3.6 to 6.1 kPa; p = 0.004).

**Fig. 3:**
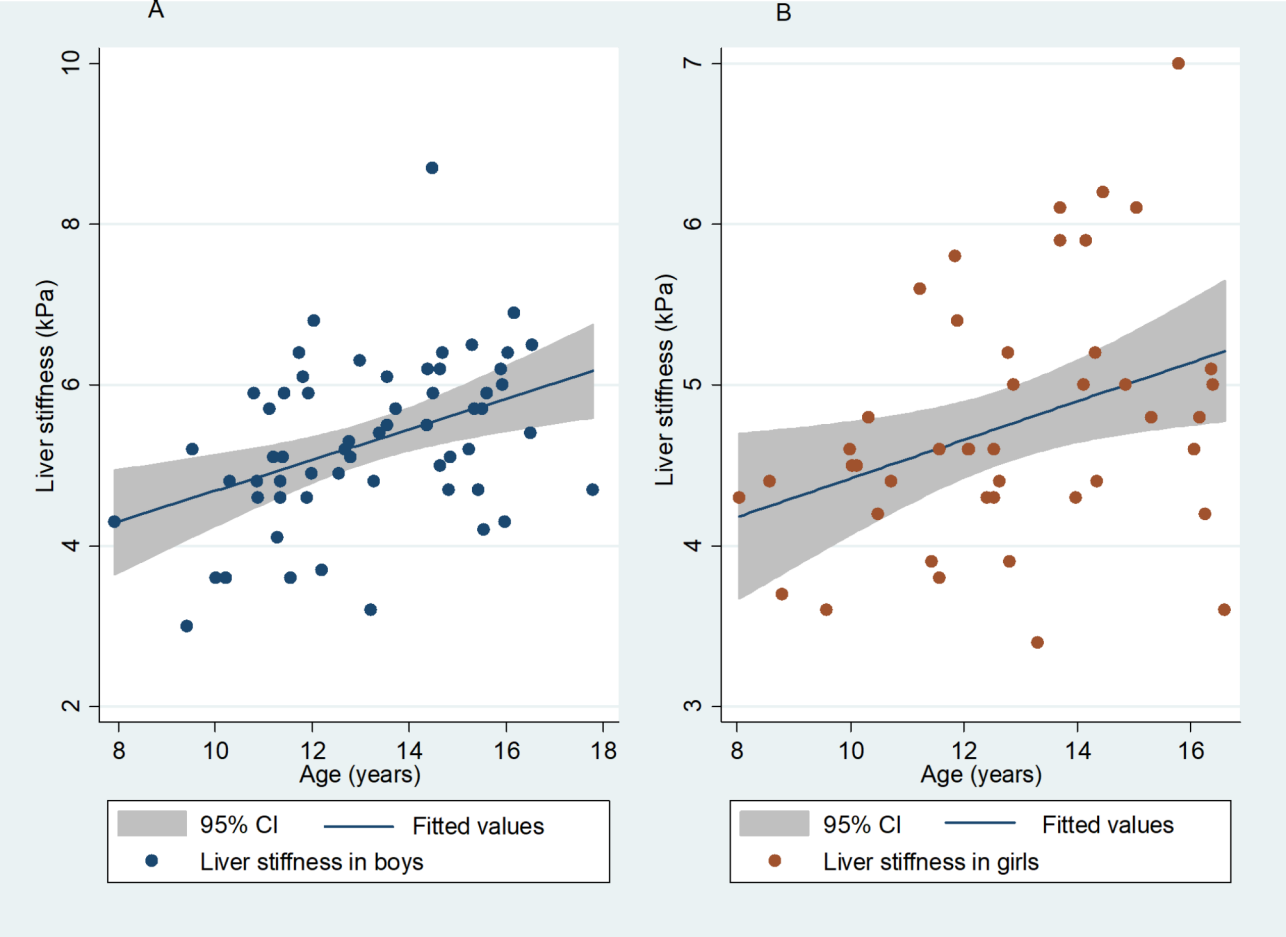
Liver stiffness median and 95% confidence interval in (A) boys and (B) girls

There was no difference in liver stiffness by ethnicity or HIV exposure status. Liver stiffness was positively correlated with age (Pearson’s r = 0.39, p < 0.001), but not waist circumference, waist-hip ratio, BMI z-score, fasting glucose, total, HDL or LDL cholesterol, triglycerides, ALT or CAP. A scatter plot and fitted values comparing liver stiffness by age in boys and girls showed higher liver stiffness in boys than girls with the difference increasing with age (Fig. 3). Liver stiffness and CAP percentiles for boys and girls are provided in Table 2.

**Table 2:** Age and sex-specific vibration-controlled transient elastography ranges for liver stiffness using the Fibroscan® M probe

Percentile	Liver stiffness in kPa				Controlled Attenuation Parameter in dB/M (boys and girls)
	Girls		Boys		
	< 12 years	≥ 12 years	< 12 years	≥ 12 years	
5th	3.6	3.6	3.6	3.7	130
25th	4.2	4.4	4.3	5.0	158
50th	4.4	4.8	4.8	5.5	179
75th	4.6	5.2	5.7	6.2	200
95th	5.8	6.2	6.1	6.9	233

### CAP results

**Fig. 4:**
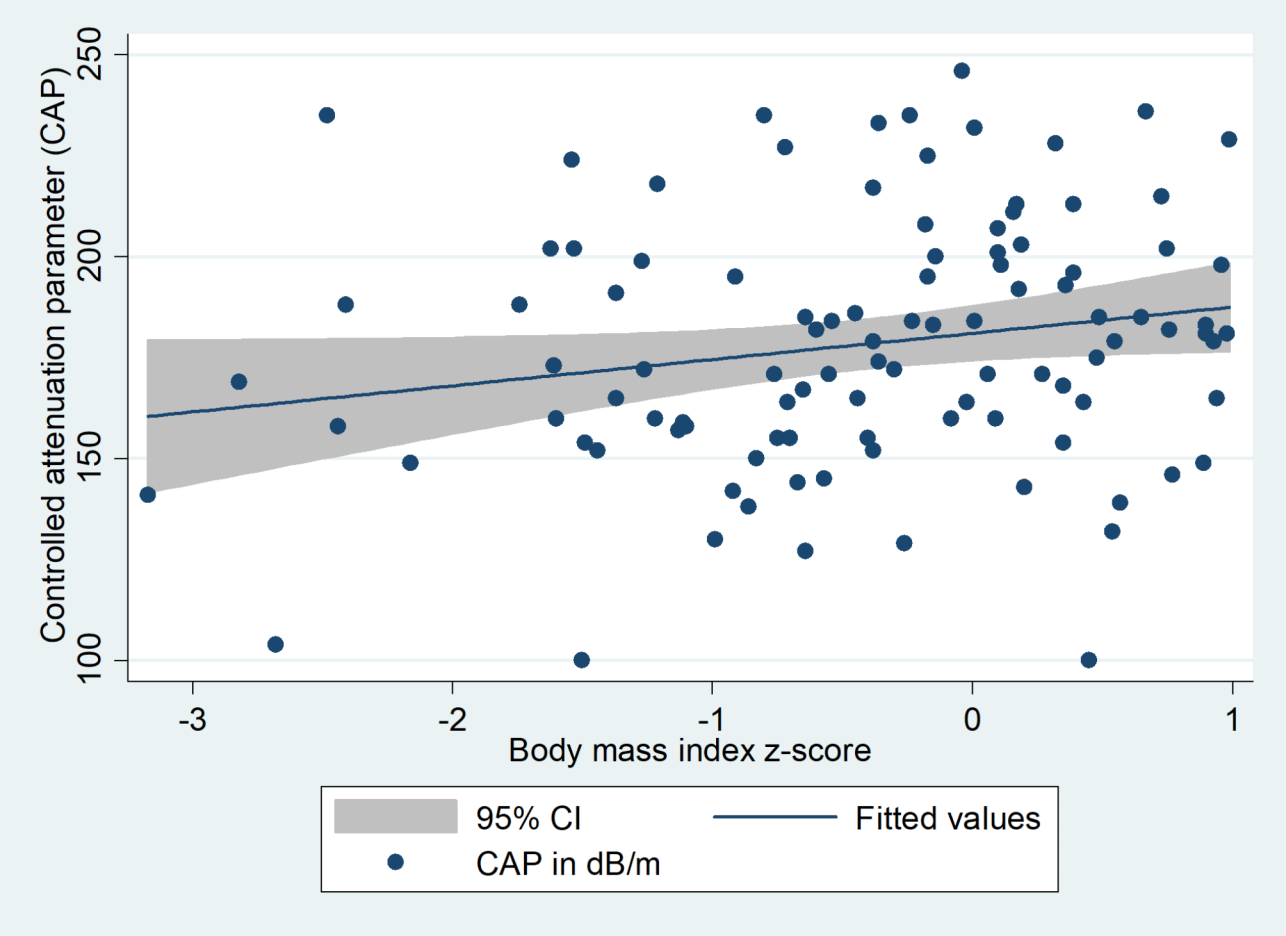
Controlled attenuation parameter and 95% confidence interval plotted against body mass index z-score

Median CAP was 179dB/m (5th to 95th percentiles 130 to 233dB/m). CAP was positively correlated with BMI z-score (Spearman’s rho = 0.2, p = 0.049), but not age, waist circumference, waist-hip ratio, fasting glucose, total, HDL or LDL cholesterol, triglycerides, ALT or liver stiffness. There was no significant difference in CAP by sex, ethnicity, HIV exposure status or pubertal status. CAP with 95% confidence intervals plotted against body mass index z-scores is provided in Fig. 4.

## Discussion

Ours is the first data providing TE values for liver stiffness and CAP in healthy African children. Although TE has been used since 2003, there are only a small number of studies of normal liver stiffness and CAP values in children. In our study of healthy South African school-aged children, liver stiffness was higher in boys than girls and increased with increasing age. CAP did not differ by age or sex but even in children who were not overweight or obese, there was a positive association between CAP and body mass index z-score.

A recent meta-analysis found that liver stiffness increases with age and that in children 3 to 18 years old, mean liver stiffness was 4.79 kPa (95% CI 4.46-5.12 kPa) with a proposed upper limit of normal of 5.56 kPa [13]. However, a limitation of this meta-analysis is that the dataset included predominantly measurements from Caucasian and Asian children. Caucasian children had significantly higher liver stiffness than Asian children, suggesting that normal values from local populations are important when interpreting liver stiffness measurements [13]. Whereas median liver stiffness values in our study of South African children were similar in girls and in boys under the age of 12 years to the median value reported in this meta-analysis, median liver stiffness in boys 12 years of age and older was higher at 5.5 kPa. The upper limit of normal in both boys and girls of all ages were higher in our study than the proposed upper limit in this meta-analysis. In a study of Egyptian living-related liver donors with normal liver histology, liver stiffness was higher in individuals with a higher BMI [20]. The authors suggested that the distribution of body fat may vary with race and that normal values should be defined for different populations. The reasons for higher liver stiffness in those with normal livers is not known and is speculative.

Although most paediatric studies found liver stiffness to increase with age, not all studies found liver stiffness to be higher in boys than girls. It has been speculated that liver stiffness is higher in males than females due to the antifibrogenic effect of oestrogen and other ovarian hormones, which inhibit extracellular matrix production by stellate cells [21, 22]. In previous studies of TE values in healthy children mean or median liver stiffness was 3.7-4.7 kPa, with the proposed upper limits of normal ranging from 6.5 to 7.9 kPa as summarised in Table 3 [9, 23–27]. There are limited available data on CAP values in healthy children. In our study, median CAP in healthy South African children was 179dB/m (5th to 95th percentiles 130 to 233), similar to previously reported studies (Table 3).

**Table 3:** Previous studies of vibration-controlled transient elastography values in healthy children

Study	Country	Dates	Total no	Age, years	Sex (M:F)	Liver stiffness kPa	CAP dB/m	Age difference	Sex difference
Goldschmidt et al.^9^	Germany	2009–2011	527	0.1–17.8 (median 6.0)	298:229	Median 4.5 (range 2.5–8.9)	NA	Liver stiffness did not vary by age	Liver stiffness did not vary between boys and girls
Engelmann et al.^23^	Germany	2009–2011	975	0–18	511:464	Median 4.7 (95th centile: 6.5)	NA	Liver stiffness increased with increasing age	Liver stiffness higher in boys than girls over 11 years
Zeng et al.^24^	China	2012–2013	436	5	236:200	Median 3.2 (5th -95th 2.0-4.8)	Median 171.5 (5th -95th 154.2-214.5)	NA	Liver stiffness did not vary between boys and girls
Lewindon et al.^25^	Australia	2011–2013	123	0.3–18 (median 8.8)	71:52	Mean 0–2 years: 3.5 ± 0.5 3–5 years: 3.8 ± 0.3 6–11 years: 4.1 ± 0.2 12–18 years: 4.5 ± 0.2	NA	Liver stiffness increased with increasing age	Liver stiffness did not vary between boys and girls
Tokuhara et al.^26^	Japan	2013–2014	139	1–18	62:77	Median (5th -95th ) 1–5 years: 3.4 (2.3–4.6) 6–11 years: 3.8 (2.5–6.1) 12–18 years: 4.1 (3.3–7.9)	Median 183 (range 112–242)	Liver stiffness increased with increasing age	Liver stiffness did not vary between boys and girls
Mjelle et al.^27^	Norway	2017–2018	243	4–17	108:135	Median 4.1 (IQR 3.5–4.6; range 2.4–11.2)	Mean 192 (range 100–296)	Liver stiffness increased with increasing age	Liver stiffness higher in boys than girls

NA = not applicable

An important factor limiting the widespread use of TE and CAP is the high cost of the FibroScan® device, with each probe entailing additional cost. Although the manufacturer recommends the S probe for children aged under 14 years or with a chest circumference less than 75 cm, our findings suggest that a standard M probe can be used in children as young as seven years. In hospitals where a FibroScan® is available, it may be a useful addition to evaluate children for possible liver fibrosis and steatosis if the CAP function is installed, and to monitor progression over time even if only an M probe is available. Although some uncertainty remains regarding the interpretation of liver stiffness and CAP measurements in children, TE has low failure rates and high intra- and inter-operator reproducibility even when used by those with little experience in the technique [28]. The safety of ultrasound is evaluated by both the thermal index to measure heating effects and the mechanical index to assess cavitation effects [29, 30]. TE is safe and well tolerated in children with no complications reported [7].

A strength of this study is that it evaluates well school-aged South African children recruited from local communities and is therefore likely to reflect the prevalence and risk factor profile in local populations of children. Children were also only included if they had a normal body mass index, had no evidence of liver disease and no metabolic risk factors. Although it is possible that including children with mildly abnormal ALT or metabolic profiles might not have changed the results obtained, we chose to be strict in our inclusion and exclusion criteria as excluding all children with any abnormal result allowed us greater certainty that the study group included only healthy children. This is, to the best of our knowledge, the first report of normal liver stiffness and CAP values in well South African children using TE. An important limitation of the study is that no liver biopsy, the currently accepted standard, was performed; however this would have been unethical in well children. This was a cross sectional study in well children and the numbers evaluated were small. Although the M probe performed well in school-aged children, TE was not evaluated in smaller children and it is likely the S probe might be necessary in these children.

## Conclusion

This is the first study reporting values for liver stiffness and controlled attenuation parameter in healthy South African children. Liver stiffness values increase with age and are higher in healthy South African boys and girls, whereas CAP values vary with BMI but not with age or sex.

## Data Availability

The dataset analysed for the current study is available from the corresponding author on reasonable request.

## Abbreviations

ALT: alanine transaminase
AST: aspartate transaminase
APRI: aspartate transaminase-to-platelet ratio
BMI: body mass index
CAP: controlled attenuation parameter
CHER: Children with HIV Early antiRetroviral
FAMCRU: Family Center for Research with Ubuntu
HDL: high-density lipoprotein
LDL: low-density lipoprotein
NHLS: National Health Laboratory Service
TE: transient elastography
